# Does Flourishing Reduce Engagement in Unhealthy and Risky Lifestyle Behaviours in Emerging Adults?

**DOI:** 10.3390/ijerph17249472

**Published:** 2020-12-17

**Authors:** Ernesta Sofija, Neil Harris, Dung Phung, Adem Sav, Bernadette Sebar

**Affiliations:** 1 School of Medicine, Griffith University, Gold Coast Campus, Southport 4222, Australia; n.harris@griffith.edu.au (N.H.); d.phung@griffith.edu.au (D.P.); b.sebar@griffith.edu.au (B.S.); 2 School of Public Health and Social Work, Faculty of Health, Queensland University of Technology, Kelvin Grove Campus, Brisbane 4059, Australia; adem.sav@qut.edu.au

**Keywords:** wellbeing–behaviour links, young adults, risk taking, lifestyle, mental health

## Abstract

Emerging adulthood is a transitional life stage with increased probability of risky and unhealthy lifestyle behaviours that are known to have strong links with premature mortality and morbidity. Wellbeing, as a positive subjective experience, is identified as a factor that encourages self-care and may steer individuals away from risky lifestyle behaviours. Investigating wellbeing–behaviour links in the emerging adult population may increase understanding of the factors that lead to, and ways to prevent, engagement in risky behaviours. This study examines the association between flourishing, that is, the experience of both high hedonic and eudaimonic wellbeing, and a broad range of risky and unhealthy lifestyle behaviours among emerging adults in Australia. A cross-sectional survey of 1155 emerging adults aged 18–25 years measured wellbeing, socio-demographics, and six groups of lifestyle behaviours surrounding substance use, physical activity, diet, sex, sun protection, and driving. Bivariate and multivariate statistics were used to analyse the data. The findings revealed that flourishing was negatively associated with more dangerous types of risk behaviours, such as driving under the influence of drugs, and positively associated with self-care behaviours, such as healthier dietary behaviour and sun protection. If enabling emerging adults to flourish can contribute to reduced engagement in risky/unhealthy lifestyle behaviours, then promoting it is an important goal for health promotion efforts not only because flourishing is desirable in its own right, but also to bring about sustainable change in behaviour. Further research is needed to inform the designs of such interventions.

## 1. Introduction

Emerging adulthood (18–29 years), particularly the age between 18 and 25, has been linked to increased probability of unhealthy and risky lifestyle behaviours, such as alcohol, tobacco and other drug use, dangerous driving, unprotected sex, and unhealthy dietary choices [1,2]. These lifestyle behaviours are associated with some of the key leading causes of premature death and disease burden in this age group [3,4]. Thus, reducing the prevalence of risky and unhealthy lifestyle behaviours among emerging adults is a priority for health promoters and governments. Largely influenced by reductionist views on health that separate mind, body, and subjective experience, a significant proportion of effort has been directed at increasing knowledge or warning about unhealthy lifestyle behaviours with at best modest success in changing behaviour [5]. Kimiecik [6] argued that without consideration of the whole individual, including subjective experiences, the efforts to change behaviour will be short-lived. Wellbeing, as a positive subjective experience, is increasingly identified as a factor that encourages self-care and may steer individuals away from risky lifestyle behaviours [2,5]. However, it is only recently that subjective experiences of the individual and their relationship to health has attracted research attention, and there is still much to learn about the complex connection between positive subjective experience of wellbeing and health behaviour [5]. 

This research will examine that connection. A number of concepts have been used to understand wellbeing. Wellbeing research has traditionally been divided into two main philosophical perspectives referred to as hedonic and eudaimonic wellbeing, which are conceptually related but distinct empirically [7,8,9,10,11]. Research suggests that these two philosophical traditions overlap and correlate, as both perspectives essentially revolve around subjective accounts of wellbeing but derive from differing operational definitions and thus denote differing aspects of it [8,11]. From a hedonic perspective, wellbeing is about maximising pleasure, positive experiences, and pleasant feelings while avoiding pain [8]. The hedonic tradition emphasises the idea of happiness, which includes immediate pleasurable experiences and also the attainment of goals [11,12,13,14]. From the eudaimonic perspective, wellbeing is viewed as a cultivation of personal strengths, actualisation of one’s inherent potential, living virtuously, and contributing to the greater good; hence, positive functioning and personal development are highlighted in this approach [8,15,16,17]. In the eudaimonic tradition, wellbeing generally relates to a sense of purpose and meaning in life and is considered to be the outcome of pursuits and fulfillment of positive personally expressive self-concordant goals [17,18].

Most researchers agree that wellbeing is a complex and multidimensional construct [7,19], which means that wellbeing stems from multiple aspects of human life. Recent research also shows that lay people hold diverse understanding of wellbeing that incorporate to varying degrees both eudaimonic and hedonic aspects [9,20]. Whilst some contention continues between hedonic and eudaimonic traditions, it is increasingly recognised that each approach denotes important aspects of wellbeing. This has led to debates regarding the need for more integrated conceptualisations and measurements of wellbeing, that incorporate both hedonic and eudaimonic aspects, and consequently, the emergence of contemporary conceptualisation and uses of flourishing as a term and a construct [8]. 

Flourishing is a term used to describe high levels of wellbeing [21]. Conceptualisations of flourishing generally capture both hedonic and eudaimonic aspects of wellbeing and as such, measures of flourishing offer a more nuanced and comprehensive assessment of wellbeing. Four prominent current conceptualisations and operational definitions of flourishing can be identified in the literature [21]. These were developed by Keyes [22], Huppert and So [23], Diener et al. [24] and Seligman [25]. Essentially, they all revolve around similar and overlapping constructs or components with some variation.

Keyes [26,27] discusses flourishing as a syndrome of hedonia (positive feelings) and eudaimonia (positive functioning), and operationalized this concept through a combination of emotional, psychological, and social wellbeing measures [22,27]. Research suggests that mental wellbeing can exist in parallel with mental ailments [22,28,29,30] and it is increasingly acknowledged that the absence of mental illness does not necessarily equate to mental health [26,28]. Keyes argued that achieving a complete state of mental health requires the presence of something positive, that is, wellbeing [22]. On this basis he introduced a two-continua model, in which mental health and mental illness are two separate but related dimensions of functioning [26,27]. The mental illness dimension relates to the extent to which a disorder is present, while the mental health dimension concerns the presence of wellbeing. The positive dimension of mental health can be defined as flourishing. To date, research on mental health and risky behaviours among emerging adults has predominantly focused on the mental illness dimension. 

Research suggests that mental health issues are some of the contributing factors to the increased uptake of risky and unhealthy lifestyle behaviours [31,32,33,34], with emerging adults being an at-risk population [35,36,37]. Self-medication and self-destructive motives are some of the explanations present in the literature for this link [38,39,40,41]. Furthermore, the turbulent and increasingly complex nature of emerging adulthood that is characterised by rapid and frequent changes in social relationships, lifestyle, employment, study, and living arrangements have been identified as factors associated with mental illness [35,36,42]. However, focusing purely on mental illness inhibits a more complete understanding of the links between mental health and lifestyle behaviours, and in turn the development of alternative or complementary approaches to address them.

If mental ailments are linked to increased risky behaviours, so may be the absence of flourishing. Indeed, research suggests that happy people live longer, and that flourishing may induce a healthier lifestyle [5,14,43]. Flourishing or higher levels of wellbeing may act as a protective factor that counteracts the need for self-medication or self-destructive behaviour as well as potentially leading to higher self-care behaviours [2,44]. Thus, high levels of wellbeing may limit the extent to which individuals engage in risky behaviours as well as increase the uptake of health promoting behaviours.

To date, most of the research that has examined the wellbeing–behaviour links among emerging adults has focused on hedonic wellbeing [45,46,47,48,49,50], with only a few studies examining the role of eudaimonic wellbeing [2,51] and only one study focusing on flourishing [52]. Even fewer studies have examined wellbeing–behaviour links among emerging adults in Australia. Further, studies that have examined the role of wellbeing in shaping behaviours related to sex, sun protection, and road behaviour are particularly sparse. Thus, the current study examines the relationship between flourishing and hedonic and eudaimonic aspects of mental wellbeing and a comprehensive range of risky/unhealthy lifestyle behaviours. Having such knowledge can broaden and deepen our understanding of the factors and ways to prevent or reduce the uptake of risky and unhealthy lifestyle behaviours. This research was conducted among emerging adults on the urban east coast of Australia through three research questions: (1) What is the association between flourishing and individual risky lifestyle behaviours? (2) What are the associations between hedonic and eudaimonic wellbeing and individual risky lifestyle behaviours? (3) What is the association between wellbeing variables and overall risky lifestyle?

## 2. Methods 

### 2.1. Study Design and Participant Recruitment

**Figure 1:**
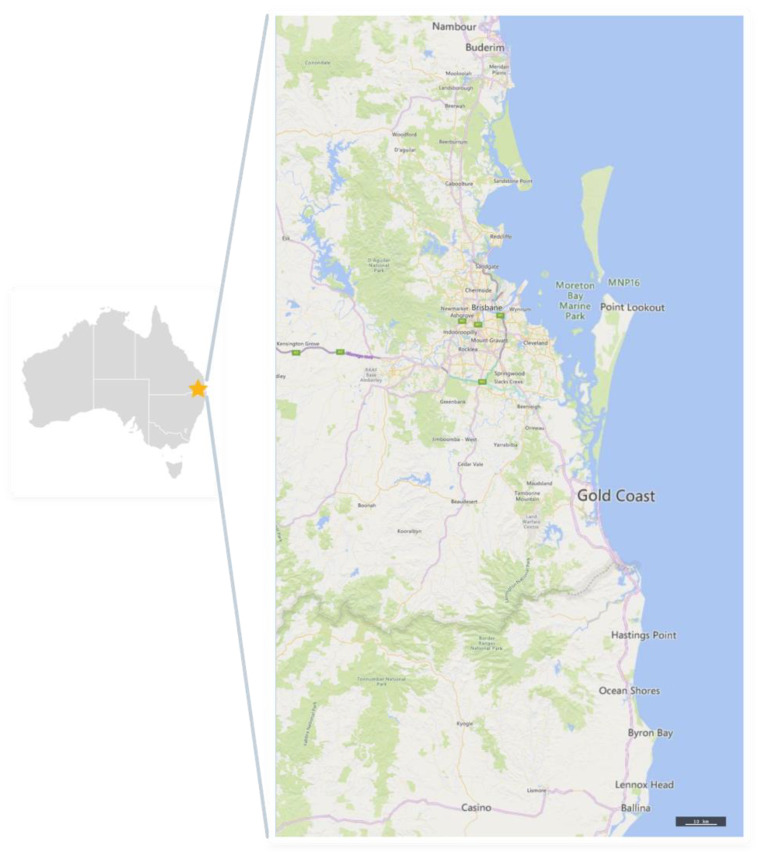
Map indicating where research participants were located (prepared using https://nationalmap.gov.au/).

This study employed a cross-sectional design using a quantitative web-based (using LimeSurvey tool) and a paper-based survey for data collection. Emerging adults who resided in South East Queensland or Northern New South Wales regions of Australia were invited to participate in this study with the majority of participants being drawn from South East Queensland (See Figure 1). The sample was selected from various community locations using convenience sampling. Participants were recruited using diverse strategies, including online advertisements on social media, posters and flyers distributed across council libraries, coffee shops, shopping centres, workplaces and similar locations and through a university research volunteers broadcast email. To diversify the sample, prospective participants were also directly approached at train stations and local colleges where they either completed the paper-based survey or were provided with the link to the web-based survey. An information sheet was provided to all participants and submission of a completed survey indicated consent to participate. The data were collected from July 2014 to April 2015. Ethics approval for this study was granted by Griffith University Human Research Ethics Committee (Ref No: PBH/50/13/HREC).

### 2.2. Measures

#### 2.2.1. Wellbeing

Mental wellbeing was assessed using the Mental Health Continuum–Long Form (MHC-LF) scale, which consists of 40 items across emotional (EWB), social (SWB) and psychological (PWB) wellbeing subscales [26,27,53]. The EWB subscale asks participants to report on the frequency of six positive emotions (e.g., feeling cheerful) during the previous 30 days on a five-point Likert scale, from none of the time to all the time. Additionally, participants rate their overall satisfaction with life by rating a single item scale from 0 to 10. The PWB scale measures six psychological wellbeing criteria: self-acceptance, personal growth, positive relations with others, purpose in life, autonomy, and environmental mastery. The SWB scale measures the five criteria of social wellbeing: social acceptance, social coherence, social actualization, social contribution, and social integration. Each criterion in these sub-scales is measured through three items where participants rate their wellbeing from strongly disagree to strongly agree. High internal consistency (Cronbach’s alpha) for each of the three sub-scales has been reported (>0.80; see, for example, Keyes [27]).

From the MHC-LF, 13 diagnostic criteria of mental wellbeing can be derived, two for hedonic (EWB) and 11 for eudaimonic (PWB and SWB) wellbeing, and wellbeing can be assessed and treated as a continuous and categorical variable [26,27]. In continuous wellbeing assessment, a high level of mental wellbeing requires participants to exhibit high levels on most measures of wellbeing, while to be diagnosed as flourishing based on categorical assessment, high levels are required on only over half of the 13 diagnostic criteria [27]. In this study, both categorical and continuous wellbeing assessments were used. For the continuous assessment, EWB, PWB and SWB scores were summated and standardized. Next, the total scores were recoded into 10-point ranges from 0 to 60 [27,54]. For categorical assessment of wellbeing, scores for each 13 diagnostic criteria were calculated, standardized, and computed into tertiles [27]. Then, dummy variables were created for diagnosis. Participants were diagnosed as flourishing if they scored highly (top tertile) on either of the two scales of EWB, and on at least six of the 11 criteria of positive functioning [53]. Participants who exhibited low levels (bottom tertile) on these criteria were categorized as languishers and those who did not meet the criteria for either flourishing or languishing were considered to experience moderate mental wellbeing [26,53]. 

Because of the small numbers of languishers (1%, *n* = 11) identified through categorical wellbeing assessment, a binary variable was created for further analyses: (1) flourishers and (0) non-flourishers (moderate and languishing mental health). Similarly, to examine the relationship between eudaimonic and hedonic wellbeing domains and lifestyle behaviours, these variables were also dichotomised into: (1) high (HH) and (0) not high (NHH) hedonic, and (1) high (HE) and (0) not high (NHE) eudaimonic wellbeing. 

#### 2.2.2. Risky/Unhealthy Lifestyle Behaviours

In total, 26 lifestyle behaviours falling under six groups were examined: (1) alcohol, tobacco and other drug use (ATODs); (2) road behaviour; (3) sun protection practices; (4) dietary behaviour; (5) unprotected sex; and (6) physical activity. These behaviours were selected due to their high prevalence among emerging adults and their associations with the leading preventable causes of mortality, morbidity, and social problems among young people. Each lifestyle behaviour was dichotomised into healthy/safe (0) and unhealthy/risky (1) categories. Lifestyle behaviour questions were drawn from existing health and risk behaviour surveillance surveys, such as the Youth Risk Behaviour survey [55] and other validated tools and questionnaires [2,56]. Questions were slightly adapted where appropriate to match the population and the Australian context. 

*ATODs’ use:* Two questions were used to measure risk associated with alcohol consumption. Participants were asked to indicate how many standard drinks they usually have on a day when they have an alcoholic drink. A standard drink guide, that explains what a standard drink is, was provided to participants to guide their responses. Australian Department of Health [57] guidelines advise against drinking more than two standard drinks on any day to reduce the risk of alcohol related disease or injury over lifetime (long-term risk). Hence, responses were partitioned into two dichotomies, safe (≤2 drinks) and risky (>2 standard drinks). The guidelines also advise against drinking more than four standard drinks in any single occasion to reduce the risk of alcohol-related injury or death arising from that occasion (short-term risk). Participants were asked to indicate on how many days during the previous month they had had 5 or more standard drinks in a row, that is, within a couple of hours, and responses were dichotomised into risky (>4 standard drinks on one day or more during the past month), and safe (0 days). For tobacco smoking, participants were asked to indicate on how many days during the past month they smoked tobacco products, even one puff. As there is no known safe threshold for tobacco use, responses were dichotomised into risky (smoked at least one day) and safe (0 days). Four questions examined drug use, with participants being asked to report on how many days during the previous month they had used marijuana, hard drugs (e.g., ecstasy, cocaine, speed, ice), sniffed something (e.g., glue, paint) or injected with a needle to get high [2]. Responses were dichotomised into risky (used at least one day) and safe (0 days).

*Road behaviour:* Participants reported on frequency of risky (distracted and impaired) driving of a car or other vehicle in the past 30 days, which refers to texting, e-mailing, or using the internet; speaking on a mobile phone held in hands; driving 10 km/h or more over the speed limit; driving while over the legal alcohol limit; and driving under the influence of drugs. A question was also included on willingness to be a passenger in a vehicle driven by a driver over the legal alcohol limit or high on drugs. Responses were partitioned into two groups, safe (never or 0 times) and risky (rarely, sometimes, often, nearly all the time or at least once). 

*Sun protection practices:* Respondents indicated how often from never to nearly all the time or always, during the last summer, on sunny days when outside for at least 15 min, they practised five behaviours; wear a hat, sunglasses, clothing that covers most of their body, use sunscreen, and seek shade [58]. Responses were dichotomised into safe (often, nearly all the time or always) and risky (sometimes, rarely, never). 

*Dietary behaviour:* Five items measured fruit, vegetable, junk food (e.g., burgers, pizza, chicken nuggets or chips from fast food outlets), sugary drinks (soft, energy or sport drinks) and sugary food (cookies, doughnuts, cake or other sweets) intake. Participants reported on how many servings of fruit and vegetable they usually ate each day. Instructions with visual examples were provided to participants to clarify what was meant by a serving. Australian Dietary Guidelines were used to classify fruit and vegetable intake as healthy (≥5 servings of vegetables and ≥2 servings of fruit) or unhealthy (<5 servings of vegetables and <2 servings of fruit) [59]. Participants also reported on how many times in the past seven days they had junk food, sugary drinks or sweets. Not consuming at all or in moderation is recommended for such items that are high in saturated fats, added salt and sugars [59]. Consumption of these items was classified as healthy (in moderation) (≤2 times) or unhealthy (≥3 times). 

*Unprotected sex:* Two items were used to measure frequency of condom use with new and casual sex partners [56]. Participants were classified as being at risk of sexually transmitted infection if they reported irregular condom use [56]. Safe category responses included always using a condom, did not have new/casual partner within the questioned timeframe, or never had sex. 

*Physical activity:* was measured using the International Physical Activity Questionnaire (IPAQ)-Short Form [60]. IPAQ assesses physical activity undertaken across comprehensive domains, taking into account lifestyle physical activity (e.g., gardening), purposive exercise, and work and transport related activity. Types of activity assessed were walking, moderate and vigorous activity with frequency (days per week) and duration (time per day) collected separately for each type of activity. The responses were calculated to profile participants as sufficiently active (Metabolic Equivalent of Task (MET), MET-min/week = 600–2999.99) and HEPA active (health enhancing physical activity level—highly active category) (MET-min/week ≥ 3000) (healthy), and insufficiently active (MET-min/week < 600) (unhealthy). Participants were also asked to indicate on how many days during the previous seven days they engaged in muscle strengthening activities/exercise. Using Australia’s Physical Activity guidelines, muscle strengthening activity was categorised as adequate (healthy) (2–7 days) and inadequate (unhealthy) (0–1 day) [61]. 

#### 2.2.3. Socio-Demographics

Data on socio-demographics including age, gender, ethnicity, religion, relationship status, living arrangements, current study and employment status, and annual personal income from all sources were collected using single-item questions drawn from existing national population-based surveys.

### 2.3. Data Analysis

Frequencies were used to quantify the prevalence of wellbeing and risky/unhealthy and safe/healthy lifestyle behaviours. Bivariate statistics using a Chi-square test was performed to examine the crude association between flourishing, hedonic and eudaimonic wellbeing, key socio-demographic factors, and each lifestyle behaviour. Multivariable Logistic Regressions were performed to examine the relationships between wellbeing and risky/unhealthy lifestyle behaviour while controlling for socio-demographic factors. 

Further, to examine the relationship between wellbeing and overall lifestyle, a Risky Lifestyle Score was calculated by summating behaviours and standardising the scores across six behaviour groups with 10 being the possible score for each group: ATODs’ use (5 behaviours*2), risky road behaviour (5 behaviours*2), unsafe sexual behaviour (2 behaviours*5), poor nutrition (5 behaviours*2), inadequate physical activity (2 behaviours*5), infrequent sun protection (5 behaviours*2). In total, 24 behaviours were included in the Risky Lifestyle Score calculations with two behaviours, namely, driving under the influence of drugs and injecting/sniffing drugs, being excluded due to small numbers. The possible Risky Lifestyle Score ranged from 0 to 60 with the higher score indicating riskier/unhealthier lifestyle. Based on possible scores, for additional analyses, the Risky Lifestyle Score was computed into tertitles: 0–19 (low risk), 20–39 (moderate), and 40–60 (high risk). A raw risky lifestyle behaviour number score that represented the number of unhealthy/risky behaviours (possible range 0–24) was also used in the analysis for comparison.

Bivariate analyses using Chi-square tests and ANOVA were performed to examine the relationship between wellbeing ranges (continuous wellbeing assessment) and overall lifestyle variables. Multivariable linear regression was used to examine the relationships between wellbeing and Risky Lifestyle Score while controlling for socio-demographic factors. 

## 3. Results

### 3.1. Participant Characteristics 

In total, 1264 surveys were completed, from which 109 cases were excluded during data cleaning. The excluded cases were those identified as outliers and those that contained extreme or suspicious values or had missing values across most of the examined variables. The final study sample comprised 1155 emerging adults aged 18–25 years (Mean age = 20.67 years), of which 74.8% were female, and 74.9% Caucasian, with 80.9% of participants reporting they were studying full-time or part-time at university. Table 1 presents a summary of participants’ socio-demographic characteristics. Not all participants answered every question (e.g., age); however, we opted not to exclude these cases from the sample as answers provided across other variables were sufficient for answering the key research questions of the study.

### 3.2. Prevalence of Flourishing and Risky Lifestyle Behaviours 

Based on categorical wellbeing assessment, 38.6% (*n* = 444) of participants were flourishers and 61.4% were non-flourishers (1%, *n* = 11 languishers; 60.4%, *n* = 695 moderately mentally well). Further, 58.2% (*n* = 672) of emerging adults exhibited high levels of hedonic wellbeing, and 47.1% (*n* = 544) exhibited high levels of eudaimonic wellbeing. The continuous wellbeing score (*n* = 924) ranged from 11.44 to 48.74 (M = 33.155; SD = 6.68). Thus, when recoded into 10-point ranges from 0 to 60, no participants’ scores fell into the ranges of 0.0–9.9 and 50.0–60.0. A small percentage of participants (3.4%; *n* = 31) fell into the lower wellbeing level range of 10.0–19.9, 80.7% (*n* = 745) fell into the middle ranges, and 16% (*n* = 148) into the second top range of 40.0–49.9. Cronbach’s alpha for each of the three sub-scales of the MHC-LF measure were >0.80 (EWB = 0.90; PWB = 0.83; SWB = 0.82), demonstrating strong internal consistency. 

Table 2 presents the prevalence of risky lifestyle behaviours. The emerging adult engagement in risky/unhealthy lifestyle behaviours varied across and within the six behaviour groups. A majority of participants did not use drugs, with the highest prevalence of use (14.1%, *n* = 163) observed for marijuana. About half of the participants reported consuming alcohol at long-term and short-term risk levels, and about one in five participants smoked tobacco at least one day in the previous 30 days. For road behaviour, the majority of drivers reported speeding (72.8%, *n* = 593) and engagement in distracted driving behaviours, with 58.3% (*n* = 475) of participants using their phones for internet and texting, and 48% (*n* = 391) speaking with a phone held in their hands. About 5% (*n* = 40) of the drivers reported driving under the influence of drugs and 10% (*n* = 99) alcohol, and 10.8% (*n* = 125) of participants were passengers of impaired drivers at least once in the previous 30 days. Sun protection practices during the previous summer varied with wearing a hat being the least (12.7%, *n* = 146) and wearing sunglasses the most (56.8%, *n* = 654) prevalent routinely-practiced behaviours. Furthermore, 32.8% (*n* = 378) of participants applying sunscreen routinely. As for dietary behaviour, only 8.3% (*n* = 93) of participants met the recommended intake of both fruit and vegetables; however, the majority of participants reported consuming, in moderation, sugary drinks (72.3%, *n* = 834), junk food (83%, *n* = 957), and sweets (57.2%, *n* = 660). Further, the majority of participants reported engaging in safe sex practices with most participants reporting regular condom use with casual (87%) and new (89.2%) sex partners. Finally, 49.9% of participants were sufficiently physically active, 26.5% met health-enhancing physical activity levels, and 23.6% reported insufficient levels of physical activity. More than half of the participants did not engage in adequate frequency of muscle strengthening activities.

The bivariate analysis on the relationship between socio-demographic factors, flourishing, high hedonic, high eudaimonic wellbeing and individual lifestyle behaviours revealed various associations across the range of variables. These results are presented as Supplementary Material.

### 3.3. The Relationship between Wellbeing and Individual Risky/Unhealthy Lifestyle Behaviours

Table 3 shows the results from Multivariable Logistic Regressions performed to examine the associations between wellbeing and engagement in each risky/unhealthy lifestyle behaviour, contrasting binary wellbeing variables (e.g., flourishers vs. non-flourishers) while controlling for all socio-demographic factors. As shown in Table 3, of six groups of behaviours, no significant relationships were found between wellbeing variables and alcohol, tobacco and other drug use (ATODs) and irregular condom use. Non-flourishers were significantly (59.1%) (OR: 1.591; 95%CI: 1.12–2.23) more likely to be physically inactive. Similarly, participants in not high hedonic (NHH) and not high eudaimonic (NHE) wellbeing level groups were 62.6% (OR: 1.626; 95%CI: 1.19–2.23) and 58.1% (OR: 1.581; 95%CI: 1.15–2.18), respectively, more likely to be physically inactive, compared to those experiencing high levels of wellbeing. No statistically significant differences were found for muscle strengthening activity. 

For road behaviour, statistically significant differences were found for four out of six examined behaviours. Non-flourishers were 217% (OR: 2.173; 95%CI: 1.34–3.53), and those in NHH and NHE wellbeing groups, 83% more likely to report being passengers of impaired drivers compared to flourishers and those in HH and HE wellbeing groups. Participants in the NHH wellbeing group were 85.7% (OR: 1.857; 95%CI: 1.14–3.02) more likely to report drink driving, while no significant differences were found between drink driving and flourishing and eudaimonic wellbeing groups. Non-flourishers (OR: 2.255; 95%CI: 1.101–5.03) and NHE (OR: 2.259; 95%CI: 1.07–4.75) wellbeing groups were 225% more likely to report driving under the influence of drugs; however, no significant differences were found between hedonic wellbeing groups. Non-flourishers were 57.1% (OR: 1.571; 95%CI: 1.09–2.26) and NHE group 65.1% (OR: 1.651; 95%CI: 1.15–2.37) more likely to report speeding. 

Significant associations were found between flourishing and two out of five investigated sun protective behaviours, namely, wearing a hat and applying sunscreen, with non-flourishers being 62.7% (OR: 1.627; 95%CI: 1.08–2.46) and 48.8% (OR: 1.488; 95%CI: 1.11–1.99), respectively, more likely to engage in infrequent uptake of these behaviours. In addition, participants in the NHE wellbeing group were 63.1% (OR: 1.631; 95%CI: 1.07–2.48) more likely to infrequently wear a hat. 

Significant associations were found between wellbeing and all dietary behaviour variables, except fruit intake. Non-flourishers were 216% (OR: 2.167; 95%CI: 1.33–3.52) less likely to meet the recommended fruit and vegetable intake when both were taken together, 55.4% (OR: 1.554; 95%CI: 1.02–2.37) less likely to meet the guidelines for vegetable intake, 80.1% (OR: 1.801, 95%CI: 1.30–2.49) more likely to consume, beyond moderation, sugary drinks, 69% (OR: 1.69; 95%CI: 1.14–2.50) junk food and 53.8% (OR: 1.538; 95%CI: 1.16–2.04) sweets. Similar relationships were found for NHH and NHE wellbeing groups and dietary behaviour, except eudaimonic wellbeing was not significantly associated with vegetable and fruit intake.

### 3.4. The Relationship between Wellbeing and Overall Risky/Unhealthy Lifestyle 

The observed total number of risky lifestyle behaviours ranged from 1 to 22 (*n* = 774; Mean = 9.693; SD = 3.51). The observed Risky Lifestyle Score ranged from 2 to 54 (*n* = 774; Mean = 22.25; SD = 8.744). The results revealed significant inverse relationships between wellbeing and risky/unhealthy lifestyles (Table 4). The proportion of participants with a high number of unhealthy/risky lifestyle behaviours (15–22 behaviours) was lowest in the high wellbeing range group (40.00–49.9), 3.6% (*n* = 4) compared to 8.9% (*n* = 69) in the lowest wellbeing range group (10.0–19.9) (*p* < 0.05). Similarly, none of the participants from the 40.00–49.9 wellbeing level range group fell into the top Risky Lifestyle Score tertile compared to 13.6% (*n* = 3) in the 10.0–19.9 wellbeing group (*p* < 0.01). Statistically significant differences were also found across wellbeing ranges and Risky Lifestyle Score Means, with the Mean of 19.70 in the highest wellbeing range and 25.18 in the lowest wellbeing range group (*p* < 0.01). 

Finally, multivariable linear regression results revealed significantly lower Risky Lifestyle Score among flourishers, HH and HE wellbeing groups, by 2.177 (95%CI: 3.56–0.79; *p* < 0.01), 1.971 (95%CI: 3.35–0.59; *p* < 0.01) and 1.780 (95%CI: 3.15–0.42; *p* < 0.05) points, respectively, compared to non-flourishers, NHH and NHE wellbeing groups adjusted for socio-demographic factors. 

## 4. Discussion

The current study examined the association between flourishing and hedonic and eudaimonic wellbeing and a broad range of lifestyle behaviours that are known to have strong links with mortality and morbidity among emerging adults. Consistent with previous research, unhealthy and risky lifestyle behaviours were found to be prevalent, with participants, on average, engaging in 10 risky/unhealthy lifestyle behaviours [62,63]. We found an inverse relationship between flourishing, hedonic, and eudaimonic wellbeing and individual risky/unhealthy lifestyle behaviours. The magnitude of the relationships was the highest for the behaviours that could be the riskiest in the short-term, namely, driving under the influence of drugs and riding in a car with an impaired driver, with non-flourishers being 217% and 225%, respectively, more likely to engage in these behaviours. The findings also revealed a significant inverse relationship between wellbeing and total risky/unhealthy lifestyle score. The findings offer insight into the relationship between wellbeing and lifestyle behaviours that could inform health promotion efforts to support health in this population.

The study findings shed light on some of the questions debated in the current literature, particularly whether it is hedonic (positive feelings, often equated with pleasure) or eudaimonic wellbeing (positive functioning, linked to purpose and meaning in life) that is more important when it comes to shaping health-related lifestyle behaviour, and more specifically, whether it varies by the behaviour in question [5]. The present study investigated hedonic and eudaimonic wellbeing aspects separately and also flourishing, which takes into account both wellbeing aspects. Further, we examined the wellbeing–behaviour links across a broad range of behaviours in one study, which has been lacking in the literature. The findings revealed that, overall, flourishing was a stronger negative predictor of individual risky/unhealthy behaviours than hedonic or eudaimonic aspects of wellbeing on their own. Flourishing was a significant negative predictor for 11, hedonic wellbeing for seven, and eudaimonic wellbeing for eight out of 26 studied individual behaviours. The observed wellbeing–behaviour links appeared to be similar for flourishing and eudaimonic wellbeing (except for vegetable and fruit intake), while hedonic wellbeing–behaviour links varied more distinctly from those observed for flourishing, with fewer behaviours reaching statistical significance. This suggests that eudaimonic wellbeing may play a more important role in shaping lifestyle behaviours among emerging adults than hedonic wellbeing. That being said, hedonic wellbeing was significantly negatively linked to drink driving, but that was not the case for flourishing or eudaimonic wellbeing. This shows that each aspect can make a distinct contribution to explaining the wellbeing–behaviour links and that these relationships vary by behaviour. Nevertheless, future research and intervention designs should consider focusing on flourishing, which has been suggested to represent a dynamic balance and interaction between positive feelings and positive functioning [8]. 

The findings suggest that interventions which nurture flourishing have the potential to contribute to healthier and less risky lifestyles among emerging adults. Wellbeing was significantly negatively associated with behaviours that could be considered to carry severe immediate consequences, such as dangerous driving, and higher likelihood of failure to practise positive lifestyle behaviours that relate to self-care (sun protection, dietary behaviour, physical activity) that most likely to impact physical health in the long-term. The finding around behaviours linked to self-care supports some of the early arguments that emerged in the literature around possible wellbeing–behaviour links made by Ryff and Singer [44]: “taking good care of yourself presupposes that your life is worth taking care of…” (p.22). Therefore, flourishers may be more inclined to take care of their health through engagement in protective and health promoting behaviours. This is an important finding and a potential avenue to explore further by public health professionals, particularly in the aftermath of the current COVID-19 pandemic. 

The finding around the negative link between wellbeing and more dangerous lifestyle behaviours is partially consistent with previous research by Schwartz et al., which investigated the associations between various aspects of wellbeing, including eudaimonic wellbeing, and illicit drug use, sexual risk taking and impaired driving, among college students [2]. In this study, associations of wellbeing were the strongest for more dangerous types of drug use, sexual behaviour, and for riding with an impaired driver [2]. Further, they found that wellbeing was associated with incidence of some sexual risk behaviour and illicit drug use, but that was not the case in our study. Schwartz et al. also found that wellbeing was associated with frequency but not the incidence of drunk/drugged driving and being a passenger in the car of an impaired driver. However, in our study wellbeing was associated with the incidence of risky road behaviours and frequency was not examined. As discussed earlier, research suggests that such risky behaviours can be linked to self-medication or self-harm motives that are incompatible with high wellbeing, and also to intensity sensation-seeking (tolerance to and a preference for high levels of stimulation) [2,64]. In addition to severe health consequences, or even death of individuals or others, behaviours like drug driving can have legal consequences. Therefore, flourishing might deter engagement in such behaviours that may jeopardise life possibilities, and flourishing emerging adults might see themselves as having more to lose [2]. In summary, the findings indicate that interventions that help achieve and sustain flourishing (e.g., through increased sense of purpose, assistance to identify and achieve self-concordant goals in life) may have potential to encourage uptake of healthier lifestyle behaviours and steer emerging adults away from risky behaviours [5,65].

Finally, the findings revealed a visible split in wellbeing–behaviour links by types of behaviours investigated, particularly when it came to potential perceived level of risk attached to certain behaviours. This points to a need to consider risk perception as a mediating variable. Wellbeing was not significantly associated with risky behaviours that could potentially be perceived to carry milder risks and to be normal given the time of increased experimentation in emerging adulthood (e.g., ATODs), with the exception of illegal hard drug use [66]. If certain behaviours, such as binge drinking are perceived as normative among emerging adults within the given context (e.g., culture where alcohol consumption is normalised), then they may be less likely to be associated with high risk and motives that are incompatible with flourishing [2]. It is important to note that in our study the use of hard drugs was significantly negatively associated with flourishing based on bivariate analysis (see Supplementary Material Table S1). However, in further analyses, after adjusting for socio-demographics, even though a clear trend was observed (OR: 1.793; 95%CI: 0.94–3.42), it did not reach statistical significance. It is possible that this was due to the small number of drug users (*n* = 64) in our sample. Considering some of the conceptual and methodological decisions in the present study may also help explain the lack of a link between wellbeing and some behaviours. We considered incidence of behaviours rather than intensity or frequency and applied a relatively strict criteria to classify behaviours into risky or safe (e.g., binge drinking once a month was classified as risky). Further, the focus was on prevalent “normal” risky/unhealthy behaviours rather than more chronic/ill behaviours, such as addictive substance use. To further advance our understanding of wellbeing–behaviour links among emerging adults, future investigations should consider including risk perception in relation to specific behaviours as a potential mediator. Health promotion efforts to achieve a shift in culture and denormalization of some of these behaviours may be an important avenue to explore, together with efforts to achieve and sustain flourishing among emerging adults.

The findings of the current research, consistent with studies conducted in other industrialised countries [2,47,67], suggest the need to support emerging adult wellbeing. Strategies are needed to address the relationship between dangerous behaviours and wellbeing. Interventions that utilise contemporary population appropriate approaches, such as social marketing, need to be developed and implemented. Many social marketing campaigns targeting emerging adults in Australia have focused on risk behaviours (e.g., “Don’t turn a night out into a nightmare” [68]). Complementary campaigns oriented on wellbeing and the importance of positive life choices are needed as part of a more holistic approach to emerging adult wellbeing. This is consistent with the recent calls to consider the whole person for sustainable change in behaviour [6].

### Limitations

Although this is one of most comprehensive studies to measure wellbeing and a range of risky behaviours in emerging adults in Australia, it has several limitations that need to be considered. First, it is bounded by the limitations of cross-sectional design, which does not allow direct testing of directionality of the relationships. High levels of wellbeing in this study were considered to precede and ward off risky/unhealthy lifestyle behaviours, but it is possible that higher levels of wellbeing can also be a result of less risky or healthier lifestyles [5,69]. That being said, and as postulated in this study, individuals with higher levels of wellbeing may be more inclined to engage in self-care activities, such as our finding around sun protective behaviours. It is also possible that individuals experience lower levels of wellbeing due to regrets related to engagement in risky behaviour, such as casual sex, that has been documented in the literature [70,71]. However, to the best of our knowledge, no published research has examined such explanations in relation to risky road behaviour. In summary, the above indicates the dynamic and likely reciprocal nature of wellbeing–behaviour links, which makes research aimed to unravel these relationships very complex [5]. Longitudinal and intervention research that focuses on promoting flourishing and measures lifestyle behaviours may offer more conclusive understanding around the strength and directionality of the relationship between wellbeing and risky/unhealthy behaviour. 

A second limitation relates to the issues associated with the use of self-reported measures, particularly those of lifestyle behaviours. Self-reported measures can be subject to individual interpretation and misreporting of behaviours together with missing responses that can potentially bias the data [72]. In the present study, the prevalence of identified behaviours is largely consistent with national statistics supporting the reliability of the self-reported measures used [63]. In addition, strategies were in place to help overcome and minimise these potential issues, for example, deliberate written and/or visual instructions to clarify questions and units in question (e.g., standard drink, serving size), and web-based surveys to encourage more honest reporting [72]. Further, while paper-based surveys were also used to help diversify the sample, no statistically significant differences were identified in reported behaviours between the modes of surveys. 

Third, the sample was recruited based on convenience. Despite the efforts to diversify the sample, the cross-section of emerging adults in this study was dominated by university students. In Australia, university students have been identified as a sub-population of emerging adults that are particularly at risk of mental health and behavioural issues [36]. Further, a reasonable sample of Vocational Education and Training students was captured to move beyond using a purely university student sample, which has been lacking in current literature, particularly in the Australian context. Nevertheless, the sample drawn represents a convenience sample of emerging adults living on the urban east coast of Australia and, therefore, the results cannot be readily generalised to the broader emerging adult population. Finally, multiple tests were performed on the data set and, accordingly, the potential for false-positive association must be acknowledged. That said, the results are consistent with previous studies in this research area.

## 5. Conclusions

The findings contribute to a growing evidence base and understanding of the link between positive subjective experiences of wellbeing and risky/unhealthy lifestyle behaviours among emerging adults. The current study identified an inverse relationship between flourishing, hedonic, and eudaimonic wellbeing and individual risky/unhealthy lifestyle behaviours among emerging adults on urban east coast of Australia. The relationships were strongest for the behaviours that are the riskiest in the short-term. Significantly lower overall risky lifestyle scores were observed among emerging adults who experienced flourishing, high hedonic and high eudaimonic wellbeing compared to those experiencing lower wellbeing. Overall, the findings indicate that wellbeing is modestly associated with lifestyle behaviours among emerging adults and serves as a factor that encourages self-care.

Addressing the high prevalence of risky/unhealthy lifestyle behaviours among emerging adults remains a challenge for health promoters worldwide. If helping emerging adults achieve and sustain flourishing can contribute even modestly to preventing engagement in risky/unhealthy lifestyle behaviours and consequent negative outcomes, then addressing the barriers to flourishing and promoting it is an important goal for health promotion interventions. This is not only because flourishing is desirable in its own right, but also to bring about sustainable change in behaviour. Further research is needed to inform the designs of such interventions and understand the extent to which they might achieve the desired goals, that are, flourishing emerging adults, reduced prevalence of risky/unhealthy lifestyle behaviours and, ultimately, happier and healthier future adults. 

## Figures and Tables

**Table 1:** Participant socio-demographic characteristics.

	N ^a^	%		N ^a^	%
**Age**			**Study status**		
18–19	454	39.4	Full-time/part-time University	929	80.9
20–21	296	25.7	Full-time/part-time TAFE/VET ^d^	142	12.4
22–23	232	20.1	Not studying	78	6.8
24–25	171	14.8			
(Missing data = 2)			(Missing data = 6)		
**Gender**			**Relationship status ^e^**		
Male	289	25.2	Not in relationship	682	59.8
Female	857	74.8	In relationship	469	40.2
(Missing data = 9)			(Missing data = 4)		
**Ethnicity**			**Living arrangements**		
Caucasian	855	74.9	Alone	42	3.7
Asian	162	14.2	Parents/other family	563	49.1
Indigenous ^b^	47	4.1	Partner	178	15.5
Other ^c^	77	6.7	Friends/housemates	363	31.7
(Missing data = 14)			(Missing data = 9)		
**Religion**			**Employment**		
No	695	62.1	Full-time permanent, contract, casual	104	9.1
Yes	425	37.9	Part-time permanent, contract, casual	594	52.0
(Missing data = 35)			Unemployed	426	37.3
			Other ^f^	19	1.7
			(Missing data = 12)		
			**Personal annual income from all sources**		
			No income	148	14.3
			$1–$12,999	407	39.4
			$13,000–$31,199	375	36.3
			$31,200 or more	104	10.1
			(Missing data = 121)		

^a^ N = 1155. Some participants did not answer every question (e.g., age). Hence, some demographic variables do not total up to 1155. ^b^ Aboriginal and/or Torres Strait Islander, Pacific Islander. ^c^ Mixed, Middle Eastern, African, Hispanic. ^d^ TAFE—Technical and Further Education, VET—Vocational Education and Training. ^e^ The not in a relationship group includes single, unattached (not committed/casual relationship) and divorced/separated; the in a relationship group includes ongoing relationship, married, de-facto. ^f^ Very sporadic casual work, holiday work, seasonal and similar.

**Table 2:** Prevalence of Risky Lifestyle Behaviours.

Alcohol, Tobacco and Other Drugs Use in the Last 30 Days					
**Marijuana**	**N**	**%**	**Alcohol (Binge Drinking > 4 Drinks per Occasion)**	**N**	**%**
Safe (0 days)	989	85.9	Safe (0 days)	612	53.1
Risky (at least 1 day)	163	14.1	Risky (at least 1 day)	540	46.9
Hard drugs (e.g., cocaine)			Alcohol (long-term risk)		
Safe (0 days)	1084	94.4	Safe (≤2 drinks)	615	53.3
Risky (at least 1 day)	64	5.6	Risky (>2 drinks)	539	46.7
Sniffed to get high or injected drugs			Tobacco smoking		
Safe (0 days)	1139	99.1	Safe (0 days)	938	81.4
Risky (at least 1 day)	10	0.9	Risky (at least 1 day)	214	18.5
**Road behaviour in the last 30 days**					
Drove car or other vehicle	N	%	Text, e-mail, internet use while driving	N	%
Yes	810	70.3	Safe (never)	340	41.7
No	342	29.7	Risky ^1^	475	58.3
Drink driving			Spoke on mobile (held in hands) while driving		
Safe (0 times)	718	87.9	Safe	424	52.0
Risky (at least once)	99	12.1	Risky	391	48.0
Drug driving			Driving 10 or more km/h over the speed limit		
Safe (0 times)	776	95.1	Safe	222	27.2
Risky (at least once)	40	4.9	Risky	593	72.8
Passenger of impaired driver					
Safe (0 times)	1029	89.2			
Risky (at least once)	125	10.8			
**Sun protection practices during last summer**					
Wear a hat	N	%	Wear protective clothing		
Safe ^2^	146	12.7	Safe	356	30.9
Risky ^2^	1004	87.3	Risky	797	69.1
Wear sunglasses			Seek shade		
Safe	654	56.8	Safe	639	55.5
Risky	498	43.2	Risky	513	44.5
Use sunscreen					
Safe	378	32.8			
Risky	774	67.2			
**Dietary behaviour**					
Usual serving intake per day			Consumption during last 7 days		
Vegetable	N	%	Soft drinks, energy or sport drinks	N	%
Healthy (≥5 serves)	126	11.2	Healthy (≤2 times)	834	72.3
Unhealthy (<5 serves)	1001	88.8	Unhealthy (≥3 times)	319	27.7
Fruit			Take away food or snacks (e.g., burgers, pizza, chips)		
Healthy (≥2 serves)	602	53.1	Healthy (≤2 times)	957	83.0
Unhealthy (<2 serves)	531	46.9	Unhealthy (≥3 times)	196	17.0
Adequate intake of both vegetable and fruit			Sweets (e.g., cookies, doughnuts)		
Yes	93	8.3	Healthy (≤2 times)	660	57.2
No	1031	91.7	Unhealthy (≥3 times)	493	42.8
**Condom use by partner type**					
In the last 12 months			In the last 3 months		
With casual partner	N	%	With new partner	N	%
Safe ^3^	993	87.0	Safe	1019	89.2
Risky ^4^	149	13.0 ^A^	Risky	123	10.8 ^B^
**Physical activity in the past 7 days**					
Muscle strengthening	N	%	Physical activity ^5^	N	%
Healthy (2–7 days)	551	48.1	Healthy ^6^	882	76.4
Unhealthy (0–1 day)	594	51.9	Unhealthy	273	23.6

^1^ Includes rarely, sometimes, often, nearly all the time or always. ^2^ Infrequent includes sometimes, rarely, never; routine includes often and nearly all the time or always. ^3^ Includes never had sex, no sex in the past 12 months, no casual/new partner or always used condom. ^4^ Includes usually (>50%), sometimes (≤50%) and never. ^5^ Includes physical activity assessment based on combination of walking, moderate-intensity and vigorous intensity activities. ^6^ Includes sufficiently active and HEPA active, which means health enhancing physical activity level—highly active category. ^A^ Irregular condom use is 15.9% when those who never had sex, or not in the past 12 months, are excluded. ^B^ Irregular condom use is 19.3% when those who never had sex, or not in the past 12 months, are excluded.

**Table 3:** Multivariable Logistic Regression results in examining the relationship between wellbeing and individual risky/unhealthy lifestyle behaviours.

	Non-Flourishers	Not High Hedonic (NHH)	Not High Eudaimonic (NHE)
**Risky lifestyle behaviour**	OR ^a^ (95% CI)	OR ^a^ (95% CI)	OR ^a^ (95% CI)
**ATODS**	N = 966	N = 966	N = 968
Marijuana	1.228 (0.83–1.8)	1.135 (0.77–1.67)	1.325 (0.90–1.95)
Hard drugs	1.793 (0.94–3.42)	1.255 (0.70–2.26)	1.599 (0.88–2.91)
Alcohol (binge drinking)	1.198 (0.90–1.59)	1.056 (0.80–1.40)	1.134 (0.86–1.50)
Alcohol (long-term risk)	1.213 (0.92–1.61)	1.286 (0.973–1.70)	1.175 (0.893–1.55)
Tobacco smoking	1.138 (0.79–1.64)	1.130 (0.79–1.61)	1.189 (0.83–1.69)
**Physical Activity**	N = 968	N = 967	N = 970
Inactive	1.591 (1.13–2.23) **	1.626 (1.19–2.23) **	1.581 (1.15–2.18) **
Insufficient muscle strengthening	1.163 (0.88–1.53)	1.022 (0.78–1.34)	1.222 (0.93–1.60)
**Road behaviour**	N = 968	N = 967	N = 970
Ride with impaired driver	2.173 (1.34–3.53) **	1.837 (1.19–2.83) **	1.833 (1.17–2.86) **
	N = 707	N = 705	N = 707
Drink driving	1.555 (0.938–2.58)	1.857 (1.14–3.02) *	1.357 (0.83–2.21)
Driving under drug influence ^b^	2.255 (1.01–5.03) *	1.386 (0.68–2.83)	2.259 (1.07–4.75) *
Speeding	1.571 (1.09–2.26) *	1.376 (0.96–1.98)	1.651 (1.15–2.37) **
Text, email, internet	1.203 (0.86–1.68)	1.098 (0.79–1.53)	1.203 (0.87–1.67)
Spoke on handheld phone	0.949 (0.69–1.31)	1.027 (0.75–1.42)	.968 (0.71–1.33)
**Infrequent sun protection**	N = 965	N = 964	N = 968
Hat	1.627 (1.08–2.46) *	1.205 (0.79–1.84)	1.631 (1.07–2.48) *
Sunglasses	1.137 (0.86–1.51)	1.304 (0.99–1.72)	1.170 (0.89–1.55)
Sunscreen	1.488 (1.11–1.99) **	1.228 (0.91–1.65)	1.307 (0.98–1.75)
Clothing	0.870 (0.65–1.17)	0.976 (0.73–1.31)	0.902 (0.67–1.21)
Seeking shade	0.862 (0.65–1.14)	0.909 (0.69–1.19)	0.854 (0.65–1.12)
**Dietary behaviour**	N = 960	N = 960	N = 960
Low fruit intake	1.230 (0.93–1.62)	1.038 (0.79–1.36)	1.227 (0.94–1.61)
Low vegetable intake	1.554 (1.02–2.37) *	1.744 (1.12–2.72) *	1.181 (0.78–1.79)
Low both fruit and vegetable intake	2.167 (1.33–3.52) **	1.801 (1.08–3.01) *	1.560 (0.96–2.53)
Soft, energy, sports drinks	1.801 (1.30–2.49) ***	1.355 (1.00–1.84)	1.735 (1.27–2.37) ***
Junk food	1.692 (1.14–2.50) **	1.792 (1.24–2.58) **	1.572 (1.08–2.28) *
Sweets	1.538 (1.16–2.04) **	1.371 (1.04–1.80) *	1.533 (1.17–2.01) **
**Irregular condom use**	N = 960	N = 959	N = 962
With new sex partners	0.887 (0.57–1.38)	1.054 (0.68–1.64)	.872 (0.56–1.36)
With casual sex partners	1.032 (0.68–1.57)	1.194 (0.79–1.81)	1.005 (0.67–1.52)

^a^ OR—odds ratio. ^b^ Not included in lifestyle score for behaviour group item balance due to small numbers. * *p* < 0.05. ** *p* < 0.01. *** *p* < 0.001.

**Table 4:** Descriptive statistics and bivariate analysis of lifestyle queried by continuous wellbeing assessment ranges.

		Wellbeing Ranges				
		10.0–19.9	20.0–29.9	30.0–39.9	40.0–49.9	
	N (%)	31 (3.4%)	263 (28.5%)	482 (52.2%)	148 (16%)	Test *p* Value
**Number of risky/unhealthy** **behaviours:**	N (%)	N (%)	N (%)	N (%)	N (%)	χ^2^ *p* < 0.05
1–7	203 (26.2)	3 (13.6)	45 (25.7)	80 (23.9)	39 (35.5)	
8–14	502 (64.9)	16 (72.7)	109 (62.3)	231 (69.0)	67 (60.9)	
15–22	69 (8.9)	3 (13.6)	21 (12.0)	24 (7.2)	4 (3.6)	
**RLS** ^1^ **tertiles:**	N (%)	N (%)	N (%)	N (%)	N (%)	χ^2^ *p* < 0.01
Bottom (0–19)	319 (41.2)	6 (27.3)	65 (37.1)	128 (38.2)	59 (53.6)	
Middle (20–39)	425 (54.9)	13 (59.1)	102 (58.3)	196 (58.5)	51 (46.4)	
Top (40–60)	30 (3.9)	3 (13.6)	8 (4.6)	11 (3.3)	0 (0.0)	
**RLS:**						ANOVA *p* < 0.01
N	774	22	175	335	110	
M; SD	22.25; 8.744	25.18; 10.48	23.14; 9.25	22.46; 8.13	19.70; 7.78	
Observed range	2–54	6–52	2–50	4–52	4–39	

^1^ RLS—Risky Lifestyle Score.

## Supplementary Materials

The following are available online at https://www.mdpi.com/1660-4601/17/24/9472/s1, Table S1: Descriptive statistics and univariate analysis of risky/unhealthy lifestyle behaviours queried by socio-demographic factors and wellbeing.
