# Supplementary material for: Does Flourishing Reduce Engagement in Unhealthy and Risky Lifestyle Behaviours in Emerging Adults?
Source: Int J Environ Res Public Health. 2020 Dec 17;17(24):9472. doi: 10.3390/ijerph17249472 (PMC7766773; doi:10.3390/ijerph17249472)
Supplement: Supplementary file 1 [file ijerph-17-09472-s001.pdf]

**Supplementary material**

**Table S1.** *Descriptive statistics and univariate analysis of risky/unhealthy lifestyle behaviours queried by socio-demographic factors and wellbeing*

| | | Alcohol, tobacco and other drugs | | | | | Physical activity | | Road behaviour | | | | | |
|---|---|---|---|---|---|---|---|---|---|---|---|---|---|---|
| **Socio-demographics** | n | Marijuana % | Hard drugs % | Binge drinking % | Long-term risk % | Smoke % | Inactive % | Insufficient muscle strengthening % | Ride with intoxicated driver % | n | Driven drunk % | Speed-ing % | Text, email, internet % | Spoke on phone % |
| **Age** | | | | | | | | | | | | | | |
| 18-19 | 454 | 10.1 | 4.6 | 42.7 | 46.8 | 13.9 | 25.6 | 54.6 | 11.7 | 320 | 10.9 | 70.5 | 48.1 | 40.1 |
| 20-21 | 295 | 18.0 | 7.1 | 49.7 | 48.0 | 19.9 | 24.0 | 52 | 10.5 | 203 | 11.8 | 77.3 | 60.1 | 50.2 |
| 22-23 | 231 | 16.0 | 6.1 | 53.2 | 48.3 | 21.6 | 21.1 | 48.9 | 10.8 | 170 | 14 | 75.4 | 69.4 | 59.1 |
| 24-25 | 170 | 15.9 | 4.7 | 44.4 | 42.1 | 24.7 | 21.6 | 48.2 | 9.4 | 121 | 13.3 | 67.8 | 66.9 | 49.6 |
| p value | | 0.014 | 0.483 | 0.041 | 0.598 | 0.006 | 0.546 | 0.376 | 0.860 | | 0.760 | 0.165 | 0.000 | 0.001 |
| **Gender** | | | | | | | | | | | | | | |
| Male | 288 | 18.4 | 7.4 | 52.9 | 52.2 | 22.5 | 20.4 | 46.5 | 15.9 | 208 | 14.4 | 74.9 | 53.6 | 47.8 |
| Female | 855 | 12.6 | 5.0 | 44.3 | 44.4 | 17.2 | 24.7 | 54.0 | 9.1 | 603 | 11.3 | 71.9 | 59.8 | 47.8 |
| p value | | 0.015 | 0.137 | 0.011 | 0.021 | 0.046 | 0.135 | 0.028 | 0.001 | | 0.230 | 0.411 | 0.120 | 0.997 |
| **Ethnicity** | | | | | | | | | | | | | | |
| Caucasian | 855 | 15.7 | 6.4 | 53.3 | 51.3 | 20.9 | 22.2 | 51.9 | 11.2 | 651 | 13.7 | 75.2 | 60.9 | 49.4 |
| Asian | 162 | 6.2 | 0.0 | 19.1 | 25.3 | 8.0 | 33.3 | 54.4 | 6.8 | 90 | 5.6 | 58.4 | 38.2 | 32.6 |
| Indigenous[a] | 47 | 17.0 | 8.5 | 53.2 | 61.7 | 21.3 | 27.7 | 48.9 | 14.9 | 33 | 9.1 | 75.8 | 69.7 | 66.7 |
| Other[b] | 77 | 11.7 | 5.3 | 27.3 | 28.6 | 11.7 | 15.6 | 51.3 | 11.7 | 36 | 2.8 | 61.1 | 47.2 | 38.9 |
| p value | | 0.013 | 0.009 | 0.000 | 0.000 | 0.001 | 0.006 | 0.904 | 0.289 | | 0.039 | 0.003 | 0.000 | 0.002 |
| **Religion** | | | | | | | | | | | | | | |
| No | 692 | 17.2 | 6.7 | 51.8 | 51.7 | 20.5 | 25.8 | 54.9 | 12.2 | 479 | 14.2 | 76.2 | 59.9 | 48.9 |
| Yes | 425 | 8.2 | 3.6 | 37.7 | 37.7 | 14.9 | 20.5 | 47.4 | 7.8 | 309 | 9.1 | 66.2 | 54.5 | 46.4 |
| p value | | 0.000 | 0.027 | 0.000 | 0.000 | 0.020 | 0.044 | 0.015 | 0.018 | | 0.031 | 0.002 | 0.136 | 0.507 |
| **Relationship status[c]** | | | | | | | | | | | | | | |
| Not in relationship | 680 | 13.7 | 6.1 | 46.8 | 46.2 | 20.1 | 22.6 | 49.0 | 11.6 | 472 | 15.0 | 70.7 | 54.4 | 46.3 |
| In relationship | 458 | 15.1 | 4.8 | 47.2 | 47.4 | 16.0 | 25.3 | 55.9 | 9.6 | 336 | 7.1 | 75.3 | 63.3 | 49.7 |
| p value | | 0.511 | 0.370 | 0.895 | 0.692 | 0.077 | 0.294 | 0.023 | 0.282 | | 0.001 | 0.149 | 0.012 | 0.338 |

| | | | | | | | | | | | | | | |
|---|---|---|---|---|---|---|---|---|---|---|---|---|---|---|
| **Living arrangements** | | | | | | | | | | | | | | |
| Alone | | | | | | | | | | | | | | |
| Parents/other family | 42 | 21.4 | 7.1 | 45.2 | 45.2 | 26.2 | 23.8 | 50.0 | 11.9 | 27 | 14.8 | 77.8 | 51.9 | 33.3 |
| Partner | 562 | 11.0 | 3.9 | 39.3 | 41.3 | 13.2 | 25.8 | 54.2 | 10.0 | 422 | 9.2 | 71.2 | 50.1 | 41.9 |
| Friends/housemates | 178 | 15.2 | 4.0 | 47.2 | 43.3 | 21.9 | 26.4 | 50.3 | 8.4 | 132 | 6.1 | 78.0 | 70.5 | 57.6 |
| p value | 361 | 17.7 | 8.1 | 58.7 | 56.7 | 23.4 | 19.3 | 49.7 | 12.9 | 230 | 20.4 | 72.2 | 66.8 | 54.8 |
| | | 0.016 | 0.039 | 0.000 | 0.000 | 0.000 | 0.114 | 0.550 | 0.355 | | 0.000 | 0.431 | 0.000 | 0.001 |
| **Study status** | | | | | | | | | | | | | | |
| University[d] | 927 | 13.8 | 5.3 | 45.1 | 46.0 | 16.2 | 23.4 | 53.0 | 9.3 | 670 | 11.2 | 72.1 | 58.1 | 46.2 |
| TAFE/VET[d] | 142 | 15.5 | 8.6 | 50.7 | 47.9 | 26.2 | 26.8 | 43.6 | 16.9 | 90 | 14.4 | 70.5 | 53.4 | 56.8 |
| Not studying | 77 | 15.6 | 2.6 | 61.0 | 52.6 | 28.6 | 21.8 | 51.3 | 17.9 | 51 | 19.6 | 82.0 | 68.0 | 52.0 |
| p value | | 0.806 | 0.137 | 0.016 | 0.513 | 0.001 | 0.612 | 0.115 | 0.003 | | 0.158 | 0.287 | 0.246 | 0.142 |
| **Employment** | | | | | | | | | | | | | | |
| Full-time[e] | 103 | 14.6 | 8.7 | 62.5 | 51.9 | 21.2 | 18.3 | 43.3 | 13.5 | 84 | 14.3 | 81.0 | 76.2 | 60.7 |
| Part-time[e] | 593 | 16.2 | 5.8 | 49.2 | 50.3 | 19.1 | 21.0 | 52.0 | 12.0 | 462 | 12.3 | 74.4 | 60.4 | 50.3 |
| Unemployed | 426 | 11.5 | 4.5 | 40.2 | 41.3 | 16.4 | 28.4 | 54.6 | 8.5 | 248 | 11.3 | 66.9 | 48.4 | 39.1 |
| Other[f] | 18 | 16.7 | 5.9 | 44.4 | 36.8 | 31.6 | 21.1 | 47.4 | 10.5 | 15 | 6.7 | 66.7 | 60.0 | 40.0 |
| p value | | 0.209 | 0.409 | 0.000 | 0.020 | 0.262 | 0.025 | 0.212 | 0.255 | | 0.809 | 0.047 | 0.000 | 0.002 |
| **Annual income** | | | | | | | | | | | | | | |
| No income | 148 | 6.8 | 2.7 | 37.2 | 35.8 | 9.5 | 25.7 | 54.1 | 8.8 | 88 | 14.8 | 66.7 | 41.4 | 35.6 |
| $1-$12,999 | 406 | 13.5 | 4.4 | 44.7 | 46.8 | 14.8 | 25.3 | 56.3 | 10.1 | 298 | 9.1 | 71.4 | 51.7 | 44.8 |
| $13,000-$31,199 | 375 | 19.7 | 8.1 | 53.1 | 53.6 | 24.9 | 19.5 | 48.3 | 13.9 | 281 | 15.3 | 75.9 | 69.5 | 56.7 |
| $31,200 or more | 103 | 14.6 | 7.7 | 62.5 | 52.9 | 26.9 | 19.2 | 43.3 | 12.5 | 87 | 13.8 | 81.6 | 70.1 | 54.0 |
| p value | | 0.002 | 0.043 | 0.000 | 0.002 | 0.000 | 0.152 | 0.036 | 0.256 | | 0.125 | 0.088 | 0.000 | 0.001 |
| **Wellbeing** | n | % | % | % | % | % | % | % | % | n | % | % | % | % |
| Flourisher | 442 | 12.6 | 3.6 | 46.4 | 46.3 | 16.1 | 16.9 | 48.5 | 6.3 | 330 | 9.7 | 70.6 | 60.3 | 50.9 |
| Non-flourisher | 705 | 15.2 | 6.7 | 47.4 | 47.2 | 20.0 | 27.8 | 54.0 | 13.6 | 487 | 13.8 | 74.2 | 56.9 | 46.0 |
| p value | | 0.227 | 0.026 | 0.742 | 0.768 | 0.100 | 0.000 | 0.072 | 0.000 | | 0.081 | 0.254 | 0.335 | 0.167 |
| High hedonic | 671 | 13.9 | 5.5 | 47.4 | 46.1 | 17.3 | 19.5 | 50.8 | 9.2 | 480 | 10.2 | 71.8 | 60.8 | 50.1 |
| Not high hedonic | 477 | 14.7 | 5.7 | 46.3 | 48.0 | 20.3 | 29.6 | 53.4 | 13.2 | 334 | 15.0 | 74.5 | 55.3 | 45.3 |
| p value | | 0.686 | 0.886 | 0.764 | 0.548 | 0.199 | 0.000 | 0.393 | 0.033 | | 0.041 | 0.402 | 0.118 | 0.182 |
| High Eudaimonic | 542 | 12.5 | 4.2 | 46.9 | 46.4 | 16.5 | 17.3 | 48.4 | 14.1 | 401 | 11.0 | 70.3 | 60.0 | 50.1 |

| | | | | | | | | | | | | | | |
|---|---|---|---|---|---|---|---|---|---|---|---|---|---|---|
| Not high Eudaimonic | 610 | 15.6 | 6.8 | 46.9 | 47.0 | 20.5 | 29.3 | 55.0 | 7.2 | 416 | 13.2 | 75.1 | 56.6 | 45.9 |
| p value | | 0.135 | 0.063 | 0.994 | 0.848 | 0.081 | 0.000 | 0.027 | 0.000 | | 0.325 | 0.124 | 0.329 | 0.227 |

| | | **Infrequent sun protection*** | | | | | **Dietary behaviour** | | | | | **Unprotected sex: Irregular condom use** | |
|---|---|---|---|---|---|---|---|---|---|---|---|---|---|
| Socio-demographics | n | Hat % | Sun glasses % | Sunscreen % | Clothing % | Shade % | Low fruit % | Low veg % | Soft, energy, sports drinks % | Junk food % | Sweets % | With new partner % | With casual partner % |
| **Age** | | | | | | | | | | | | | |
| 18-19 | 452 | 86.9 | 50.6 | 70.9 | 67.1 | 44.2 | 47.1 | 89.6 | 29.5 | 15.6 | 42.5 | 7.8 | 8.2 |
| 20-21 | 295 | 89.8 | 47.1 | 69.8 | 67.6 | 41.4 | 50.0 | 89.6 | 27.4 | 16.6 | 44.3 | 12.1 | 14.5 |
| 22-23 | 231 | 87.4 | 32.5 | 60.2 | 71.9 | 46.8 | 39.6 | 87.7 | 27.3 | 16.9 | 43.7 | 13.9 | 16.0 |
| 24-25 | 171 | 83.6 | 32.2 | 62.6 | 73.7 | 47.4 | 50.0 | 86.9 | 23.5 | 21.2 | 39.4 | 12.4 | 19.4 |
| p value | | 0.279 | 0.000 | 0.015 | 0.298 | 0.523 | 0.087 | 0.726 | 0.520 | 0.432 | 0.765 | 0.061 | 0.001 |
| **Gender** | | | | | | | | | | | | | |
| Male | 286 | 86.7 | 55.4 | 80.5 | 64.2 | 49.8 | 49.5 | 88.4 | 41.9 | 26.3 | 41.9 | 12.2 | 15.3 |
| Female | 856 | 87.5 | 39.4 | 62.6 | 70.7 | 42.3 | 45.7 | 89.1 | 22.8 | 13.6 | 43.2 | 10.3 | 12.2 |
| p value | | 0.728 | 0.000 | 0.000 | 0.041 | 0.026 | 0.277 | 0.754 | 0.000 | 0.000 | 0.731 | 0.377 | 0.169 |
| **Ethnicity** | | | | | | | | | | | | | |
| Caucasian | 853 | 87.1 | 39.6 | 65.3 | 71.3 | 47.2 | 46.5 | 87.5 | 27.9 | 14.5 | 42.4 | 12.6 | 14.7 |
| Asian | 162 | 87.6 | 56.5 | 70.8 | 66.7 | 34.8 | 53.2 | 95.6 | 21.6 | 24.1 | 46.3 | 3.2 | 5.7 |
| Indigenous[a] | 47 | 85.1 | 48.9 | 78.7 | 66.0 | 48.9 | 41.3 | 91.3 | 42.6 | 40.4 | 36.2 | 10.9 | 17.4 |
| Other[b] | 77 | 89.6 | 53.2 | 74.0 | 51.9 | 31.2 | 39.0 | 85.5 | 27.3 | 13.0 | 37.7 | 8.0 | 9.2 |
| p value | | 0.893 | 0.000 | 0.078 | 0.004 | 0.002 | 0.168 | 0.022 | 0.042 | 0.000 | 0.482 | 0.005 | 0.010 |
| **Religion** | | | | | | | | | | | | | |
| No | 693 | 90.5 | 41.4 | 67.3 | 70.5 | 45.8 | 47.7 | 87.8 | 27.2 | 16.3 | 43.8 | 12.0 | 14.9 |
| Yes | 422 | 83.4 | 46.6 | 67.8 | 66.0 | 41.6 | 45.4 | 90.0 | 27.8 | 18.2 | 41.0 | 8.3 | 9.3 |
| p value | | 0.000 | 0.088 | 0.847 | 0.121 | 0.169 | 0.459 | 0.268 | 0.836 | 0.413 | 0.383 | 0.056 | 0.007 |
| **Relationship status[c]** | | | | | | | | | | | | | |
| Not in relationship | 677 | 86.7 | 45.1 | 69.8 | 67.5 | 47.1 | 45.0 | 87.9 | 26.7 | 18.5 | 44.1 | 13.9 | 17.5 |
| In relationship | 459 | 88.7 | 40.5 | 64.1 | 71.7 | 40.5 | 49.3 | 90.0 | 28.4 | 14.9 | 41.1 | 6.2 | 6.6 |
| p value | | 0.326 | 0.129 | 0.042 | 0.134 | 0.028 | 0.152 | 0.278 | 0.542 | 0.127 | 0.329 | 0.000 | 0.000 |

| | | | | | | | | | | | | | |
|---|---|---|---|---|---|---|---|---|---|---|---|---|---|
| **Living arrangements** | | | | | | | | | | | | | |
| Alone | 42 | 88.1 | 52.4 | 59.5 | 64.3 | 50.0 | 38.1 | 88.1 | 35.7 | 23.8 | 31.0 | 17.1 | 19.5 |
| Parents/other family | 559 | 87.1 | 46.9 | 72.4 | 63.6 | 41.0 | 48.7 | 89.8 | 26.3 | 17.6 | 45.8 | 7.6 | 9.2 |
| Partner | 178 | 87.6 | 30.3 | 57.9 | 69.7 | 41.6 | 48.0 | 85.6 | 35.6 | 16.4 | 41.8 | 6.3 | 8.0 |
| Friends/housemates | 362 | 87.6 | 43.1 | 64.6 | 78.0 | 50.0 | 44.8 | 88.7 | 24.3 | 15.2 | 39.5 | 16.6 | 20.5 |
| p value | | 0.995 | 0.001 | 0.001 | 0.000 | 0.040 | 0.429 | 0.514 | 0.023 | 0.493 | 0.103 | 0.000 | 0.000 |
| **Study status** | | | | | | | | | | | | | |
| University | 927 | 87.4 | 43.3 | 65.7 | 70.7 | 42.1 | 48.2 | 89.0 | 25.9 | 14.9 | 43.3 | 9.8 | 11.7 |
| TAFE/VET | 139 | 88.5 | 52.9 | 77.9 | 62.4 | 57.9 | 40.4 | 85.6 | 35.5 | 26.2 | 36.2 | 16.9 | 18.2 |
| Not studying | 78 | 84.6 | 25.6 | 64.1 | 62.8 | 47.4 | 40.3 | 90.9 | 34.6 | 25.6 | 47.4 | 11.7 | 19.5 |
| p value | | 0.708 | 0.001 | 0.015 | 0.064 | 0.002 | 0.116 | 0.419 | 0.022 | 0.000 | 0.190 | 0.040 | 0.023 |
| **Employment** | | | | | | | | | | | | | |
| Full-time[d] | 104 | 84.6 | 29.8 | 72.1 | 67.3 | 43.3 | 37.3 | 90.2 | 31.7 | 17.3 | 46.2 | 15.4 | 19.2 |
| Part-time[d] | 589 | 89.0 | 42.0 | 67.3 | 70.1 | 47.4 | 46.5 | 88.5 | 26.8 | 18.2 | 43.7 | 11.1 | 14.3 |
| Unemployed | 426 | 85.2 | 48.6 | 66.2 | 68.3 | 41.3 | 50.4 | 89.3 | 26.6 | 14.8 | 40.7 | 8.6 | 9.5 |
| Other[e] | 19 | 100.0 | 36.8 | 57.9 | 68.4 | 26.3 | 42.1 | 73.7 | 42.1 | 21.1 | 47.4 | 15.8 | 10.5 |
| p value | | 0.086 | 0.004 | 0.555 | 0.905 | 0.096 | 0.110 | 0.195 | 0.350 | 0.517 | 0.663 | 0.168 | 0.028 |
| **Annual income** | | | | | | | | | | | | | |
| No income | 147 | 89.8 | 55.8 | 71.4 | 71.6 | 43.5 | 51.7 | 86.5 | 25.7 | 14.2 | 33.8 | 6.2 | 6.9 |
| $1-$12,999 | 406 | 84.2 | 42.6 | 67.5 | 67.5 | 42.9 | 46.4 | 90.5 | 25.4 | 16.3 | 41.5 | 8.2 | 9.7 |
| $13,000-$31,199 | 373 | 90.3 | 39.7 | 66.4 | 72.5 | 48.5 | 46.6 | 86.6 | 28.8 | 18.4 | 42.1 | 15.1 | 19.9 |
| $31,200 or more | 104 | 85.6 | 31.7 | 70.2 | 66.3 | 45.2 | 40.4 | 90.4 | 31.7 | 16.3 | 41.3 | 15.4 | 17.3 |
| p value | | 0.053 | 0.001 | 0.680 | 0.365 | 0.433 | 0.370 | 0.283 | 0.501 | 0.682 | 0.338 | 0.002 | 0.000 |
| **Wellbeing** | n | % | % | % | % | % | % | % | % | % | % | % | % |
| Flourisher | 442 | 83.9 | 39.3 | 61.9 | 70.7 | 46.3 | 43.3 | 86.4 | 22.8 | 13.3 | 37.5 | 11.1 | 12.9 |
| Non-flourisher | 703 | 89.3 | 45.6 | 70.3 | 68.1 | 43.6 | 49.2 | 90.4 | 30.8 | 19.1 | 46.0 | 10.6 | 13.2 |
| p value | | 0.008 | 0.035 | 0.003 | 0.359 | 0.376 | 0.052 | 0.040 | 0.004 | 0.012 | 0.005 | 0.817 | 0.868 |
| High hedonic | 668 | 86.2 | 39.8 | 65.2 | 69.9 | 45.0 | 46.3 | 87.1 | 25.9 | 14.6 | 40.4 | 10.9 | 12.9 |
| Not high hedonic | 476 | 89.1 | 48.0 | 69.8 | 68.3 | 44.0 | 48.0 | 91.2 | 30.0 | 20.2 | 46.0 | 10.7 | 13.4 |
| p value | | 0.152 | 0.005 | 0.099 | 0.586 | 0.745 | 0.584 | 0.032 | 0.140 | 0.016 | 0.061 | 0.886 | 0.783 |
| High Eudaimonic | 542 | 84.5 | 39.2 | 63.9 | 70.5 | 45.9 | 43.7 | 87.8 | 23.2 | 14.0 | 38.3 | 11.1 | 13.2 |
| Not high Eudaimonic | 608 | 89.8 | 46.8 | 70.1 | 67.9 | 43.3 | 49.7 | 89.7 | 31.6 | 19.7 | 46.7 | 10.4 | 12.9 |

| | | | | | | | | | | | |
|---|---|---|---|---|---|---|---|---|---|---|---|
| p value | 0.007 | 0.010 | 0.025 | 0.328 | 0.393 | 0.041 | 0.316 | 0.002 | 0.012 | 0.004 | 0.710 | 0.887 |

Significant findings are highlighted in red

*includes sometimes, rarely, never

[a]Aboriginal and/or Torres Strait Islander, Pacific Islander

[b] mixed, Middle Eastern, African, Hispanic

[c] not in a relationship group includes single, unattached (not committed/casual relationship) and divorced/separated; In a relationship, includes ongoing relationship, married, de-facto

[d] includes full-time and part-time

[e]permanent, contract, casual

[f]very sporadic casual work, holiday work, seasonal and similar
